# Beyond traditional treatments: vagal nerve stimulation for painful temporomandibular disorders

**DOI:** 10.3389/fpain.2026.1860767

**Published:** 2026-07-23

**Authors:** Rania Nuwailati, David P. Darrow, Donald R. Nixdorf

**Affiliations:** 1 Department of Diagnostic and Biological Sciences, Division of TMD and Orofacial Pain, School of Dentistry, University of Minnesota, Minneapolis, MN, United States; 2 Department of Oral Medicine, School of Dentistry, University of Washington, Seattle, WA, United States; 3 Department of Neurosurgery, Medical School, University of Minnesota, Minneapolis, MN, United States; 4 Department of Radiology, Medical School, University of Minnesota, Minneapolis, MN, United States

**Keywords:** electrical stimulation, facial pain, neuromodulation, pain management, treatment resistant

## Abstract

**Introduction:**

Chronic temporomandibular disorders (TMD) can progress to refractory chronic pain in a subset of patients, for whom standard multimodal treatments often provide limited relief. Emerging neuromodulation therapies-particularly non-invasive vagal nerve stimulation (nVNS)—suggest a potential non-pharmacologic adjunct within multimodal strategies for managing persistent symptoms. This case series explores the use of non-invasive nVNS in conjunction with a multimodal treatment strategy for managing refractory pain in patients with chronic TMD.

**Methods:**

A retrospective review at the University of Minnesota TMD, Orofacial Pain and Dental Sleep Medicine Clinic, evaluated patients with chronic TMD pain treated with a multimodal treatment plan which included GammaCore nVNS device, by comparing their baseline data to outcomes at their most recent follow-up.

**Results:**

Three patients (mean age 44.3 ± 23.3 years) with refractory TMD pain were identified as having received nVNS therapy using the GammaCore device. Treatment duration ranged from 4.6 to 18.2 months and was self-administered three to four times per day. All three patients reported improvements in pain intensity, pain-related disability, and psychosocial functioning, in the context of a multimodal treatment plan that included nVNS.

**Discussion:**

Our limited clinical experience observed improvements across multiple domains relevant to patients with chronic TMD in the context of a multimodal treatment plan that included nVNS.

IRB approval status: Approved STUDY00024420.

## 1 Introduction

Chronic temporomandibular disorders (TMD) represent a heterogeneous group of musculoskeletal conditions involving the temporomandibular joint (TMJ), masticatory muscles, and associated structures (1). Pain trajectories among TMD patients vary; approximately 15% develop chronic pain lasting for at least three months (2), 20% of which progress to refractory chronic pain (R-TMD) (1, 3). R-TMD is characterized by persistent or worsening symptoms which are unresponsive to standard multimodal therapies (4).

Chronic TMD is typically managed with a multimodal approach, including pharmacologic and behavioral interventions, therapies, and occasionally surgery (5). Self-care and oral analgesics are first-line strategies and play critical roles in chronic pain management, in addition to the pharmacologic approach. However, these therapies often provide inadequate relief, contributing to significant individual and societal burdens for patients with R-TMD (6). Moreover, prolonged use raises concerns about adverse effects (7). The advanced management strategies of R-TMD are further complicated by comorbidities (8). Evidence suggests impaired endogenous pain modulation contributes to persistence and treatment resistance. Psychosocial factors, including cognitive difficulties, pain-related rumination, depressive symptoms, and reduced quality of life, also contribute to pain maintenance and functional impairment (9).

Neuromodulation encompasses implantable and non-implantable technologies, electrical or chemical, for the purpose of improving quality of life and functioning of humans (10, 11). It is applied across conditions such as chronic pain, psychiatric disorders, and neurological diseases, offering a targeted alternative to -or potentially enhancing—the pharmacologic treatments with different side effect profiles (11). Vagal nerve stimulation (VNS), first introduced in the late 1990s for refractory epilepsy (12), was FDA-approved in 1997 for refractory epilepsy and in 2005 for treatment-resistant depression (13). Traditional VNS requires surgical implantation, but non-invasive VNS (nVNS) has since been developed to stimulate the vagus nerve at the cervical level (in the neck) (14, 15) or via auricular branches (around the ear) (16, 17). The FDA cleared non-invasive VNS in the late 2010's for cluster headache and migraine (18). Unlike transcutaneous electrical stimulation (TENS) which provide short-term localized pain relief, VNS targets afferent pathways to induce long-term modulation of central nervous system activity, offering broader and longer-lasting effects on neurological, psychiatric, and autonomic functions.

The gammaCore device is a portable, handheld tool used for (nVNS) therapy (19, 20). It delivers mild electrical pulses through the skin on the neck to stimulate the vagus nerve, modulating pain signaling and restoring autonomic balance. Each self-administered adjustable treatment lasts approximately two minutes. Designed for ease of use, gammaCore offers a convenient, drug-free option for managing refractory pain conditions.

Since 2016, multiple studies have investigated various treatment options for R-TMD, from minimally invasive techniques to surgery, highlighting the potential of nVNS as an adjunctive therapy due to the condition's complex biopsychosocial nature (21). nVNS is already approved for migraine and depression; common TMD comorbidities. Its mechanism including mood regulation, anti-inflammatory effects via the cholinergic anti-inflammatory pathway, autonomic rebalancing, and enhanced endogenous pain inhibition, are highly relevant to chronic TMD pathophysiology (22–24). This case series examines nVNS use in conjunction with a multimodal strategy for managing R-TMD pain.

## 2 Methods

Retrospective chart review included patients seen at the TMD, Orofacial Pain, and Dental Sleep Medicine Clinic at the University of Minnesota School of Dentistry between October 31, 2021, and August 21, 2023. The study was approved by the University of Minnesota Institutional Review Board (IRB No. STUDY00024420).

This case series included patients aged 18 years or older who had at least one definitive TMD diagnosis, confirmed through clinical examination and supported by a score greater than 3 on the TMD Pain Screener—a validated tool for identifying TMD-related pain—and who experienced refractory facial pain. Both male and female patients were included. All patients received nVNS as part of their treatment for managing refractory pain. Patients were excluded if they did not present with refractory facial pain and if nVNS was not part of their treatment regimen.

Data were extracted from the TMD Pain Screener (25), demographic questionnaire, and standardized instruments. of the Diagnostic Criteria for Temporomandibular Disorders (DC/TMD) protocol, routinely used by clinicians and completed by patients during clinic visits:

- Axis I (Clinical Assessment)
- Axis II (Biobehavioral Assessment): the Graded Chronic Pain Scale (GCPS) (26), Jaw Functional Limitation Scale-8 (JFLS-8) (27), Generalized Anxiety Disorder 2-item (GAD-2) Patient Health Questionnaire 2-item (PHQ-2) (1), and Catastrophizing and Expectation (Catast/Expect) (28).

Additionally, patients completed Headache Impact Test (HIT-6) (29), Perceived Stress Scale-4 (PSS-4) (30), Sleep Disturbance measures (31), and European Quality of Life 5 Dimensions 5 Level Version (EQ-5D-5L) (32). Clinical outcomes were assessed at baseline (office visit 1, when nVNS was initiated) and at follow-up visits (office visits 3–6).

### 2.1 Statistical analysis

Descriptive statistics were used to summarize the data. Frequencies and percentages were calculated for each instrument to determine the percent change in patient scores between the baseline (when nVNS was prescribed) and the final follow-up visit. A Wilcoxon Test was conducted using Jamovi Project 2024 to assess the significance of changes between the first and last visits for each patient. This nonparametric test accommodated the small sample size (*n* = 3) and the lack of normal distribution assumptions, making it more suitable for this exploratory analysis. The test compared paired observations for each outcome measure; statistical significance set at *p* < 0.05. No adjustment for multiple comparisons, consistent with the exploratory nature of the study. The Wilcoxon test is reported for descriptive and exploratory purposes only. Given the sample size of *n* = 3, results are mathematically insufficient for inferential conclusions and should not be interpreted as such.

## 3 Results

All diagnoses were confirmed by an experienced, board-certified orofacial pain specialist (DRN) with over 20 years of clinical experience. Patients were included in this case series if they met these criteria: (1) confirmed diagnosis of at least one chronic TMD condition, established during a follow-up appointment; (2) prescription of nVNS using the FDA-approved GammaCore® device as part of the treatment plan for refractory facial pain; and (3) a documented history of prior treatment with medications that were either ineffective or minimally effective in managing symptoms. Given the inadequate response to previous therapies including pharmacological interventions, nVNS was introduced as an alternative therapeutic option.

### 3.1 Patients characteristics

Three Caucasian patients were included, including two females and one male. All patients were diagnosed with Bilateral TMJ arthralgia and myalgia and experienced persistent TMD-related pain unresponsive to conventional therapies. The follow-up period ranged from 4.6 to 18.2 months, during which each patient attended 3–6 follow-up visits. In addition to their individualized treatment plans, all three patients were prescribed daily, bilateral self-administered nVNS therapy using the GammaCore® device, targeting the Vagus Nerve (Cranial Nerve X) running from the brainstem down through the neck. Demographic characteristics, diagnostic details, treatment regimens, and medication histories are presented in Table 1.

**Table 1:** Patients characteristics.

	Case (1)						Case (2)						Case (3)					
A-Demographics:																		
Age y/o	71						28						34					
Gender	F						M						F					
Marital Status	Married						Never Married						Never Married					
Ethnicity	Not Hispanic or Latino						Not Hispanic or Latino						Not Hispanic or Latino					
Race	White						White						White					
Highest level of education	Professional or post-graduate level						College graduate						College graduate					
Household income	$60,000–$79,999						$20,000–$39,999						$0–$19,999					
B-Clinical Diagnosis and Prescribed Treatment:																		
Medication History	*Pain medications*:						*Other medications:*						*Other medications:*					
	Gabapentin (600 mg)						Cotempla 25mg						Drospirenone–ethinyl estradiol					
	Venlafaxine (250 mg)						Ketamine 80 mg						Lisdexamfetamine					
	Flexeril (5 mg)						Zolpidem tartrate 3,5 mg						Duloxetine (60 mg)					
	Acetaminophen						Mirtazapine 40mg						Cyclobenzaprine					
	Ibuprofen						Melatonin 5 mg						Psyllium fiber					
	*Other medications:*												Magnesium glycinate					
	Levothyroxine												Methylated folate					
	Trazodone												Fish oil					
	Fluticasone												Vit D					
	Ziprasidone												Melatonin					
	Atomoxetine												Medical cannabis					
	hydrochloride																	
Number of Office Visits (course of treatment in months)	6 (12 m)						4 (18.2 m)						3 (4.6 m)					
Office visits attended	1	2	3	4	5	6	1	2	3	4	5	6	1	2	3	4	5	6
	√	√	√	√	√	√	√	√	√	√	–	–	√	–	√	–	–	–
Diagnosis	Bilateral TMJ arthralgia and myalgia:						Bilateral TMJ arthralgia and myalgia:						Bilateral TMJ arthralgia and myalgia:					
	Myofascial pain of the muscle of mastication and cervical muscles						Myofascial pain of the muscle of mastication and cervical muscles						Myofascial pain of the muscle of mastication and cervical muscles Episodic tension-type headache					
							Idiopathic orofacial dystonia											
Treatment plan	nVNS (Gamma core)						nVNS (Gamma core)						nVNS (Gamma core)					
							Ketamine (12 m)											
							Botulinum injection											
Description	2 min twice						2 min twice						2 min twice					
	3–4 time/day						3–4 time/day						3–4 times/day					
							40 mg lozenges											
							3 times/day											
							Xeomin in masster temporalis & posterior cervical muscles											

F, female; M, male; *m*, month; y/o, years old; TMJ, temporomandibular Joint; nVNS, non-invasive vagus nerve stimulation.

### 3.2 Detailed case description

#### 3.2.1 Case 1

A 71-year-old female presented with bilateral TMJ region pain; it began and was more pronounced on the right side. The pain radiated to the masseter, sternocleidomastoid (SCM), and shoulder muscles. She described the pain as throbbing, with an intensity ranging from 5 to 7 out of 10 and reported intermittent exacerbations throughout the day with frontal headaches (rated 5/10) and right ear pain. Pain was alleviated with heat packs, shoulder exercise, and TMJ massage, but was aggravated by stress and right shoulder activity, particularly work-related tasks.

The patient also reported sleep deprivation, averaging fewer than 7–8 h of sleep per night due to pain. She had previously undergone physical therapy (PT) sessions for jaw, and shoulders and neck pain. She uses heat on her jaw and shoulders twice daily (morning and evening) providing temporary symptom relief; however, pain typically worsened by the end of the day. Her occupation as a dental hygienist required prolonged forward head posture, which exacerbated her symptoms.

Her *medical history* included hypothyroidism and arthritis which lead to left knee and ankle replacement surgery. Psychiatric history included borderline personality disorder, bipolar disorder, depression, and anxiety.

Clinical examination revealed bilateral TMJ tenderness on palpation without joint sounds. Muscle palpation demonstrated tenderness in bilateral masseter and upper trapezius, as well as right temporalis, right temporalis tendon, and right SCM,

The patient was diagnosed with bilateral TMJ arthralgia, myalgia and tension type headache.

Initial management included patient education on the masticatory system function and a home jaw self-care program consisting of heat and ice therapy, pain-free diet, bilateral chewing, identifying and decreasing daytime oral habits, TMJ massages, and three jaw exercises (stretching, rotation, and relaxation). Botulinum toxin injections were recommended for the right jaw, neck, and shoulder. nVNS using the GammaCore device was initiated at a frequency of three to five times daily; beginning on the right (more painful) side and then alternating bilaterally.

During follow-up over 12-month period of GammaCore use, the patient initially reported increased pain intensity on workdays and fluctuations related to sleep quality and stress which gradually improved over time. Additionally, interventions included trigger point injections to the masseter and temporalis muscles, botulinum toxin injections at the previously recommended sites, and use of a maxillary flat-plane splint. By the third follow-up visit, her pain decreased to 2/10, prompting temporary discontinuation of nVNS for one month. At her subsequent visit, she reported a recurrence of pain with intensity of 9/10. The treatment plan was modified to resume nVNS, along with continued botulinum toxin injections, additional trigger point injections, and oral appliance adjustments.

#### 3.2.2 Case 2

A 28-year-old male presented with sharp, stabbing pain involving the masseter and cervical muscles, progressing from 6/10 to 9/10 in intensity. He reported facial spasms and a pulling sensation along with difficulty relaxing his facial muscles. The pain had been present since childhood and worsen over the preceding five years. He also reported daily, constant headaches that were predominantly dull but intermittently piercing in quality. Stress was a major exacerbating factor and frequently triggered headache episodes. The patient reported to be diagnosed with chronic sleep initiation insomnia, mild delayed cardician rhythm, and non-restorative sleep He noted that facial spasms were precipitated by chronic sleep disturbance and insomnia.

His medical history included Ehlers-Danlos syndrome, anxiety, and depression. Prior PT, NSAID, night guards, and home self-care measures were ineffective. Patient reported difficulty swallowing pills and was limited to dissolvable or sublingual formulations. He also reported prior use of cyclobenzaprine, with development of tolerance.

Clinical examination revealed bilateral TMJ tenderness on palpation, with clicking noted in the left TMJ. Muscle palpation demonstrated tenderness of the bilateral masseter and temporalis muscles. Mandibular parafunctional movements were observed and appeared to be exacerbated by insomnia.

The patient was diagnosed with bilateral TMJ arthralgia, myalgia, left TMJ disc displacement with reduction, and oromandibular dystonia.

Management included PT sessions targeting the masseter, temporalis, frontalis, SCM, and suboccipital muscles, along with a prescribed home program including exercises, self-massage, and self-care program consisting of heat and ice therapy. Botulinum toxin injections were administered. At follow-up, the patient reported a 30% overall improvement. While botulinum injections offered temporary relief on the left side (lasting approximately 90 days), jaw pain intensified (8–9/10), particularly on the right. Headaches changed in quality from sharp to dull. The patient reported reaching a plateau with the current treatment. Clonidine was initiated at 0.2 mg three times daily. Despite dosage modifications, clonidine was discontinued after three months for lack of efficacy. PT, home care, and botulinum injections every three months were continued. One month later, the patient began daily nVNS using the GammaCore device (three to five times per day for two minutes for 18 months) and was prescribed sublingual ketamine (40 mL). After four months of this combined therapy, his pain decreased from 7 to 9/10 to 4/10, with notable improvements in psychosocial well-being.

#### 3.2.3 Case 3

A 34-year-old female presented with generalized dental pain rated 6/10, which began following the loss of her orthodontic retainer. She described intense pressure affecting all teeth, heightened sensitivity to thermal stimuli (particularly in the canines), and bilateral facial pain with masseter tightness radiating to the head and retroauricular regions. Her jaw pain was daily, constant, throbbing, and aching in nature (rated 8/10), and worsened with jaw function and chewing throughout the day. The patient reported prior use of an orthodontic retainer but denied use of a night guard or oral splint for pain management. She also reported intermittent jaw clicking and popping, with partial symptom relief following prior TMJ-directed physical therapy. She also reported jaw locking during dental procedures and exhibited parafunctional habits including clenching, grinding, and tongue pressing. The patient experienced frequent headaches (10–30 days per month), described as sharp and aching with an intensity of 9/10, without a history of migraines. Additional symptoms included left shoulder spasms, neck tightness radiating to the arm, fibromyalgia flare-ups, sleep disturbance, fatigue, and difficulty managing head and neck pain despite a pain management program.

Her medical history was notable for hypermobility and joint instability, previously treated with PT since childhood. Medical comorbidities included fibromyalgia, chronic congestion, suspected mast cell disease, post-traumatic stress disorder, sleep disorder, depression, anxiety, attention-deficit/hyperactivity disorder, irritable bowel syndrome, scalp and inner ear psoriasis, human papillomavirus infection, premenstrual dysphoric disorder, and a prior neck injury related to violin use. A review of systems revealed widespread symptoms such as dizziness, joint pain, and hypersensitivities.

Clinical examination revealed tenderness of the bilateral TMJs, masseter, temporalis, SCM, splenius capitis, suboccipital muscles, trapezius, temporalis tendon, and lateral pterygoid muscles. Intraoral examination revealed bilateral Linea alba, tongue scalloping, and a Mallampati class IV airway. The Beighton hypermobility score was 2/9. Dental examination excluded odontogenic sources of pain. Cervical side-bending elicited pain with mildly restricted rotation.

The patient was diagnosed with bilateral TMJ arthralgia and myalgia, left TMJ disc displacement with reduction and intermittent locking, myofascial pain (MFP) of the masticatory and cervical muscles, and episodic tension-type headaches secondary to TMJ pain.

The multidisciplinary treatment plan included home self-care, heat and cold therapy, pain-free diet, bilateral chewing, oral habit awareness and reduction, PT, jaw exercises, a maxillary stabilization splint, and daily nVNS (three to five times daily for two minutes).

During follow-up visits, the patient reported progressive improvement. Jaw pain in the preauricular area decreased from 8/10 to 6/10 (20% improvement), and later to 4/10 (75% total reduction). Jaw function and pressure sensitivity improved. The patient reported complete resolution of headaches since initiating treatment and a decrease in oral parafunctional behaviors. Neck pain and cervical stability also showed marked improvement.

### 3.3 Pain characteristics

**Figure 1:**
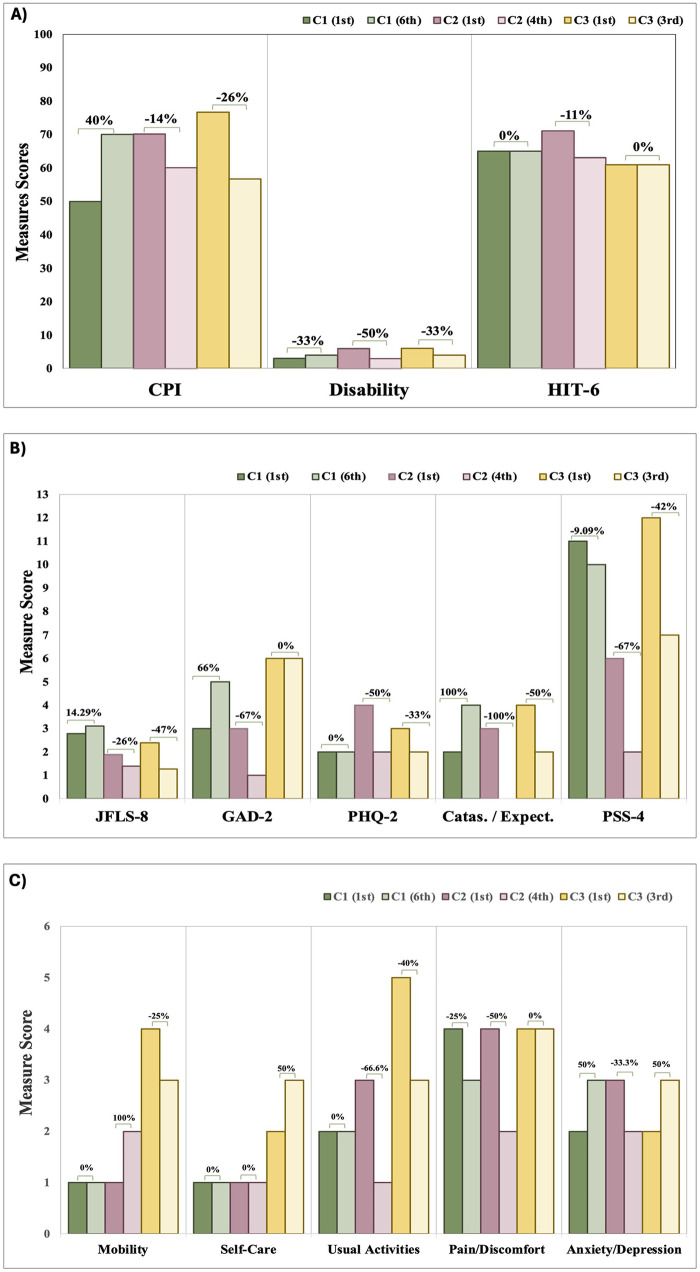
**Patient-Reported Outcome Measures Tracking Clinical Changes Over Time.** The data points reflect changes in scores between the first visit (baseline) and the last visit for each patient-specifically, **Case 1 (C1)** across 6 visits, **Case 2 (C2)** across 4 visits, and **Case 3 (C3)** across 3 visits. (**A**) **Pain Characteristics:** Visualizing reductions in patient-reported pain across three domains: CPI (Characteristic Pain Intensity), Disability, and HIT-6 (Headache Impact Test). (**B**) **Psychosocial and Behavioral Characteristics:** Reductions in functional and psychological burden from baseline to last visit across multiple scales: JFLS-8 (Jaw Functional Limitation Scale-8), GAD-2 (Generalized Anxiety Disorder 2-item), PHQ-2 (Patient Health Questionnaire 2-item), Catast/Expect (Pain Catastrophizing and Treatment Expectation), and PSS4 (Perceived Stress Scale-4). (**C**) **Health-Related Quality of Life:** Individual shifts in the EQ-5D-5L (European Quality of Life 5-Dimensions 5-Level Version), showing improvements across all 5 dimensions from baseline to the final visit.

The TMD Pain Screener revealed a 43% overall reduction in pain scores, with the most notable improvement observed in Case 3 (Tables 2, 3). The GCPS, which evaluates both pain intensity and its interference with daily activities, showed substantial improvement in Cases 2 and 3; however, Case 2 was concurrently receiving sublingual ketamine (a potent NMDA receptor antagonist), which may confound the observed reduction in characteristic pain intensity. Specifically, the Characteristic Pain Intensity (CPI) decreased by 14%–26%, while the pain-related disability score dropped by 33%–50%, reflecting enhanced functional capacity and reduced pain burden. Importantly, the overall GCPS pain grade improved in both cases, shifting from the most severe level (Grade IV) to a moderate level (Grade III), indicating meaningful clinical benefit (Figure 1).

**Table 2:** Patients responses.

Office Visit		Case (1)			Case (2)			Case (3)			Mean (SD) (Baseline)	Mean (SD) Follow-Up
		1	6	%	1	4	%	1	3	%		
Pain Characteristics:												
TMD Pain Screener Score		4	5	25	5	5	0	7	4	−43	5.3 (1.5)	4.6 (0.6)
GCPS	CPI	50	70	40	70	60	−14	76.67	56.67	−26	65.55 (13.8)	62.2 (6.9)
	Disability	3.00	4.00	33.33	6.00	3.00	−50	6.00	4.00	−33	5 (1.7)	3.6 (0.6)
	Grade	III	III	–	IV	III	–	IV	III	–	–	–
HIT-6	Score	65	65	0	71	63	−11	61	61	0	56.67 (5)	6 (2)
	Interpretation	VSI	VSI	–	VSI	VSI	–	VSI	VSI	–	–	–
Psychosocial and behavioral characteristics:												
Catast/Expect	Score	2	4	100	3	0	−100	4	2	−50	3 (1)	2 (2)
	Q1	Very good	Very good	–	Good	Good	–	Very good	Good	–	–	–
PSS-4	Score	11	10	−9.09	6	2	−67	12	7	−42	9.6 (3.2)	6.3 (4.04)
JFLS-8	Score	2.72	3.11	14.29	1.89	1.39	−26	2.39	1.28	−47	2.3 (0.4)	1.9 (1.02)
GAD-2 PHQ-2	GAD-2 Score	3	5	66.67	3	1	−67	6	6	0	4 (1.7)	4 (2.6)
	PHQ-2 Score	2	2	0.00	4	2	−50	3	2	−33	3 (1)	2 (0)
	Q2	Not difficult at all	Not difficult at all	–	Some what difficult	Not difficult at all	–	Extremely difficult	Very difficult	–	–	–
	Q3	Very Good	Very Good	–	Fair	Fair	–	Poor	Poor	–	–	–
Assessment of Quality of Life and Sleep:												
Quality of sleep	Q4	Most of the time	Most of the time	–	None of the time	None of the time	–	None of the time	None of the time	–	–	–
Sleep Disturbance Grade	Score	47.9	52.2	8.98	60.4	55.3	−8	64.9	66.1	2	57.73 (8.8)	57.8 (7.3)
	Interpretation	Non-Slight	Non-Slight	–	Moderate	Mild	–	Moderate	Moderate	–	–	–
EQ-5D-5L	Score	11,242	11,233	–	11,343	21,122	–	42,542	33,343	–	–	–
	Q5	83	80	−3.6	57	65	14	49	54	10	–	–
Follow-Up	Q6	–	No change	–	–	Better and a definite improvement	–	–	Moderately better	–	–	–
	Q7	–	No change	–	–	–	–	–	–	–	–	–

(Percentage) %, percent of change in scores from baseline score to their last follow-up visit; TMD, temporomandibular disorders; CPI, characteristic pain intensity; GCPS-Grade, Graded Chronic Pain Scale; HIT-6, Headache Impact Test; Catast/Expect, Catastrophizing and Expectation; JFLS-8, Jaw Functional Limitation scale; GAD-2/PHQ-2, Generalized Anxiety Disorder 2-item and Patient Health Questionnaire 2-item; PSS4, Perceived Stress Scales; EQ-5D-5L, European Quality of Life 5 Dimensions 5 Level; EQ-5D-5L interpretation: #Mobility, #Self Care, #Usual Activities, #Pain/Discomfort, #Anxiety/Depression; Q1, I feel that the treatment outcome for my face pain will turn out; Q2; If you checked off any of the problems for the questions above, how difficult have these problems made it for you to do your work, take care of things at home, or get along with other people; Q3, In general, would you say your health is; Q4, In the last 30 days, do you feel refreshed or rested when you awaken after a night's sleep; Q5, To help people say how good or bad a health state is, we have drawn a scale (rather like a thermometer) on which the best state you can imagine is marked 100 and the worst state you can imagine is marked 0. Please indicate on this scale how good or bad your own health is today, in your opinion; Q6, Since beginning treatment at this facility, how would you describe the change (if any) in ACTIVITY LIMITATIONS, SYMPTOMS, EMOTIONS, and OVERALL QUALITY OF LIFE, related to your painful condition; Q7, In a similar way to Q6, please select the number below, that matches your degree of change since beginning care at this clinic; VSI, very severe impact.

**Table 3:** Within-Subject changes in outcome measures (Wilcoxon test).

Instrument	Statistic	*P* value	Mean difference	SE difference	95% CI		Effect Size
					Lower	Upper	
TMD Screener	2.00^a^	1.000	1.000	1.202	1.00	1.00	0.333
CPI	3.50	1.000	3.373	12.019	−20.00	20.00	0.167
Disability	5.00	0.500	1.500	1.202	−1.00	3.00	0.667
HIT-6	1.00^b^	1.000	8.000	2.667	NaN	NaN	1.000
JFLS-8	1.50^a^	1.000	0.000	0.577	0.00	0.00	0.000
GAD-2	1.50^a^	1.000	0.000	1.155	0.00	0.00	0.000
PHQ-2	3.00^a^	0.371	1.500	0.577	1.50	1.50	1.000
Catast/Expect	4.50	0.586	0.922	1.528	−2.00	3.00	0.500
PSS4	6.00	0.250	3.500	1.202	1.00	5.00	1.000
Sleep Disturbance	2.50	1.000	−1.058	2.963	−5.00	5.00	−0.167

Results are exploratory only. Sample size (*n* = 3) precludes inferential interpretation. CPI, characteristic pain intensity; HIT-6, Headache Impact Test; JFLS-8, Jaw Functional Limitation scale; GAD-2/PHQ-2, Generalized Anxiety Disorder 2-item and Patient Health Questionnaire 2-item; Catast/Expect, Catastrophizing and Expectation; PSS4,: Perceived Stress Scales-4. *Note. Hₐ μ Measure 1—Measure 2 ≠ 0.*

a 1 pair(s) of values were tied.

b 2 pair(s) of values were tied.

However, limited changes on headache symptoms were reported, with only an 11% reduction in HIT-6 score reported in Case 2 (Tables 2, 3). Despite persistent facial and orofacial pain within the past 30 days reported by all three patients, improvements were noted in the number of days pain interfered with daily activities. On average, patients reported 10.5 ± 8.1 days of activity limitation. Notably, Case 2 showed a reduction from 20 to 1 day (95% improvement), and Case 3 from 30 to 4 days (86.7% improvement).

### 3.4 Psychosocial and behavioral characteristics

The most pronounced change on psychosocial and behavioral outcomes, was observed particularly in Cases 2 and 3 (Tables 2, 3). Notable improvements were observed across several domains commonly affected in chronic pain and TMD populations. Catastrophizing scores, which reflect exaggerated negative responses to pain, decreased by 50%–100%, indicating a substantial shift in patients' cognitive appraisal of their condition. Treatment expectation scores also improved markedly, suggesting increased optimism and perceived effectiveness of the intervention.

Perceived stress levels, measured by PSS-4, were reduced by 9%–67%, reflecting improved stress regulation and potentially enhanced coping mechanisms. Functional limitations, as measured by the JFLS-8, decreased by 26%–47%, indicating improved ability to perform daily activities involving jaw function.

In addition, symptoms of anxiety and depression, assessed via the GAD-2 and PHQ-2, were reduced by 33%–67%, highlighting the positive impact of nVNS on emotional well-being.

### 3.5 Assessment of quality of life and sleep

nVNS therapy had minimal impact on sleep quality, with only a modest 8% reduction in the sleep disturbance score observed in Case 2. This suggests that, despite its effectiveness in other domains, nVNS was not notably beneficial for addressing sleep-related issues in these patients.

Health-related quality of life assessed using the EQ-5D-5L instrument—a standardized tool evaluating five dimensions of well-being—showed variable improvements across cases. Notably, Case 3 demonstrated the most noticeable gains in mobility, suggesting enhanced physical function. Both Cases 2 and 3 reported improvements in the ability to perform usual daily activities, reflecting better functional independence.

Reductions in pain and discomfort were most evident in Cases 1 and 2, aligning with the overall improvement in TMD-related symptoms. Additionally, Case 2 showed improvement in the anxiety/depression dimension.

## 4 Discussion

The findings from this case series suggest that the addition of nVNS to the treatment plan may represent a promising approach to enhance the therapeutic benefit for patients with chronic, refractory TMD pain. All three patients reported reductions in pain intensity, improved functional ability and enhanced psychosocial well-being. These outcomes align with emerging evidence supporting the analgesic and neuromodulatory effects of vagal nerve stimulation in chronic pain conditions. Notable improvements included a 43% reduction in the TMD Pain Screener score for Case 3, decrease in CPI by up to 26%, and reductions in pain-related disability scores of up to 50%. These results align with previous research suggesting that nVNS modulates pain perception pathways and induces analgesia through central and peripheral mechanisms (33–35). The precise mechanisms remain under investigation, however, both animal and clinical studies have identified multiple pathways involved. Centrally, VNS has been shown to inhibit nociceptive transmission within the spinothalamic and spinoreticular tracts and to suppress spinal trigeminal neuron activity, with lasting inhibitory effects (36–38). Besides, it influences key structures involved in descending pain modulation, including the nucleus tractus solitarius (NTS), locus coeruleus (LC), periaqueductal gray (PAG), and nucleus raphe magnus (NRM) (39, 40). Functional imaging further demonstrated that VNS enhances connectivity within brain networks associated with pain inhibition and engages serotonergic, noradrenergic, and opioidergic pathways (10).

Beyond analgesia, psychosocial improvements were observed. Patients showed reductions in pain catastrophizing (up to 100%), perceived stress (up to 67%), and symptoms of anxiety and depression (up to 67%), particularly in Cases 2 and 3. These findings suggest that nVNS may enhance emotional regulation and stress resilience, critical factors in the biopsychosocial model of chronic pain. Supporting evidence from reviews and meta-analyses indicates that nVNS has potential as an adjunctive treatment for mood disorders, including depression, with a favorable safety profile compared to invasive VNS (41, 42). In clinical populations, nVNS has also been shown to improve quality of life and reduce depression scores in patients with persistent postural-perceptual dizziness, a condition often comorbid with anxiety and depression (43). Mechanistically, nVNS is believed to regulate autonomic nervous system balance, increase norepinephrine secretion, and enhance heart rate variability, all of which likely contribute to improved mood and stress resilience (41, 44). Despite these promising findings, most studies are small or preliminary. Large-scale, controlled trials are required to confirm these effects and clarify their mechanisms, particularly in relation to psychosocial distress beyond mood disorders (41, 44, 45).

Of note, Case 1 showed worsening anxiety symptoms, highlighting the importance of accounting for individual differences in psychological status, pain chronicity, and coping strategies. Future research should investigate tailored approaches that integrate psychological support alongside neuromodulation.

In contrast, nVNS showed minimal impact on sleep quality, with only an 8% improvement in sleep disturbance observed in a single case. This suggests that sleep outcomes in TMD may require multimodal interventions, including behavioral, pharmacological, and lifestyle strategies. Adjunctive treatments specifically targeting sleep may be necessary to achieve comprehensive improvements in quality of life.

The therapeutic effects of nVNS are likely multifaceted. In addition to modulating nociceptive transmission and central pain pathways, nVNS may attenuate neuroinflammation via anti-inflammatory cytokine regulation. For instance, transcutaneous VNS (tcVNS) has been shown to reduce pro-inflammatory cytokines such as TNF-α, IL-1β, and IL-8 while increasing levels of the anti-inflammatory cytokine IL-10 in healthy individuals (46–48).

These immunomodulatory effects may contribute to the mood-enhancing and pain-relieving benefits observed in this series. Our findings are consistent with prior studies demonstrating the efficacy of both invasive and non-invasive VNS for chronic pain conditions such as fibromyalgia and migraine (10). Moreover, recent research indicates that nVNS can modulate trigeminal nociceptive processing, providing a plausible mechanism for its benefits in orofacial pain disorders such as TMD (49). Given that high baseline pain intensity has been identified as a risk factor for poor recovery in TMD over time (50). The potential effect of interventions like nVNS on pain reduction, and disability may have important implications for long-term outcomes.

Despite these encouraging findings, this study has several limitations. First, the small sample size, of three patients renders this work purely exploratory and proof-of-concept; no clinically generalizable conclusions can be drawn. The retrospective design and absence of a control group further limit the generalizability of the results and introduce the possibility of placebo effects. Second, it is not possible to isolate the therapeutic effect of nVNS from the concurrent interventions each patient received. The observed improvements must therefore be interpreted as occurring within the context of these multimodal treatment plans, rather than attributable to nVNS alone. Third, treatment duration varied widely across cases, ranging from 4.6 to 18.2 months, representing a substantial confound that limits consistent baseline-to-follow-up comparisons across the cohort. Combined with variability in adjunctive therapies and baseline psychosocial profiles, this heterogeneity further constrains cross-case interpretation.

. Future randomized controlled trials (RCTs) with larger sample sizes are needed to establish the efficacy, safety, and optimal dosing protocols for nVNS in the treatment of refractory TMD. Further investigation into its long-term effects, particularly on sleep disturbances, is warranted to provide a more comprehensive understanding of its therapeutic potential.

## 5 Conclusion

This case series suggests pain reduction, enhanced functional abilities, and improved psychosocial well-being in three patients with chronic refractory TMD receiving multimodal treatment plans that included nVNS. Given the small sample, retrospective design, and concurrent interventions these findings are hypothesis-generating only. Further research is needed to elucidate the mechanisms underlying nVNS's effects and to explore standardized protocols for its potential application in TMD management.

## Data Availability

The original contributions presented in the study are included in the article/Supplementary Material, further inquiries can be directed to the corresponding author.
